# Performance optimization of planar photonic crystal bound states in the continuum cavities: mitigating finite-size effects

**DOI:** 10.1007/s12200-025-00147-5

**Published:** 2025-03-14

**Authors:** Ran Hao, Bilin Ye, Jinhong Xu, Yonggang Zou

**Affiliations:** 1 State Key Laboratory on High Power Semiconductor Lasers, Changchun University of Science and Technology, Changchun, 130022 China; 2 College of Optical and Electronic Technology, China Jiliang University, Hangzhou, 310018 China

**Keywords:** Bound states in the continuum, High-quality factor, Graded photonic crystals, Electrical pumped laser

## Abstract

Bound states in the continuum (BICs) offer a promising solution to achieving high-quality factor (*Q* factor) cavities. However, finite-size effects severely deteriorate the BIC mode in practical applications. This paper reports the experimental demonstration of an electrically pumped 940 nm laser based on optimized BIC cavity, achieving a high *Q* factor of up to $1.18 \times 10^4$ even with finite photonic crystal footprint, which is two orders of magnitude larger than un-optimized BIC design. Two strategies have been systematically investigated to mitigate finite-size effects: reflective photonic crystal cavity design and graded photonic crystal cavity design. Both methods significantly improve the *Q* factor, demonstrating the effectiveness of preserving BIC characteristics in finite-sized photonic crystal cavities. In addition, the reflective boundary photonic crystal design is fabricated and experimentally characterized to demonstrate its lasing characteristics. The fabricated laser exhibits single-mode operation with a signal-to-noise ratio of 38.6 dB. These results pave the way for future designs of BICs with finite size in real applications, promoting the performance of BIC-based integrated lasers.

**Graphical Abstract:**

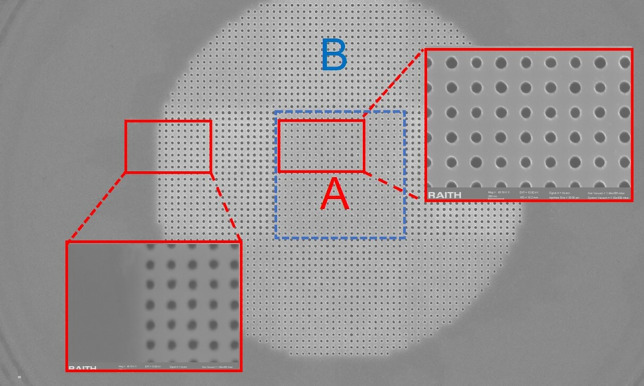

## Introduction

Photonic crystal cavity has received extensive attention due to its large freedom to tail the cavity behaviors, thus facilitate laser cavity designs [1–3]. However, due to the reflective Fabry–Pérot (FP) cavity in photonic crystal, the high quality factor (*Q* factor), narrow linewidth and high output power are difficult to achieve at the same time [4, 5]. Moreover, the design of high performance photonic crystal lasers often requires the incorporation of multiple sets of lattice nested together or the use of irregular geometric unit cells [5–7]. These approaches typically involve highly complex fabrication processes with stringent precision requirements, making the fabrication challenging. In recent years, a novel laser based on Bound States in the Continuum (BIC) has garnered attention. Unlike traditional FP cavity lasers, BIC-based photonic crystal lasers require only a single lattice but intrinsically support an infinite large *Q* factor without out-of-plane radiation loss, resulting in simplified fabrication complexity and high *Q* values [8].

BIC refer to localized states that can stably exist in open space without any boundary constraints in the continuous domain [9–11]. In contrast to conventional FP cavities, a perfect BIC laser supports zero coupling between the BIC mode and the radiation modes, thus can eliminate leakage. Theoretically, this not only yields an infinite quality factor *Q*, but also significantly reduces the linewidth and pumping threshold [10]. However, the perfect BIC mode requires photonic crystal with infinite lattice size which is not possible to be realized in real applications [11]. Kodigala et al. firstly reported the finite size effect in BIC laser by compared the performances of ${8\times 8}$, ${10\times 10}$, ${16\times 16}$, ${20\times 20}$ array size laser cavity, and found that *Q* factor drops rapidly as the laser array size decreases [9]. In order to maintain the high *Q*, Zhao et al. use 3 cm photonic crystal to maintain the BIC mode [12]. There is a few research that pay attention to how to mimic the BIC behavior with a finite size photonic crystal design [13, 14]. A reciprocal-space matching technique is proposed but requiring fine tuning of the thickness of photonic crystals which adds difficult in fabrication [13]; dipole model and dispersion model are proposed to analyze the finite size of sub-wavelength resonators [14]. So far, a simple but effective way to achieve BIC in finite size device is still unsolved and greatly appreciated. In addition, current research in BIC lasers mostly rely on optical pumping lasers that do not need optoelectronic conversion [9, 11, 15, 16]. The reported electric pumping lasers with BIC are only achieved in Terahertz range, e.g., ${\sim 3}$ THz [6, 17], so far an electric pumped BIC laser in optical domain has not been reported yet, revealing the difficulties in appropriate optoelectronic design and sub-wavelength fabrication.

In this paper, an electronic pumped 940 nm laser based on bound states in the continuum via photonic crystal cavity was experimentally demonstrated for the first time, achieving a quality factor of up to ${1.1776\times 10^{4} }$. We have systematically investigated two approaches to improve the BIC effect in finite size, reflective photonic crystal cavity and graded photonic crystal cavity. Our results proved an effective yet simple method to improve the *Q* factor in a finite size photonic crystal (*N*=11) by two orders of magnitude, while reducing the line width of laser and increasing the confinement of single laser mode. The design philosophy based on BIC not only achieves a high *Q* factor but also optimizes multiple performance metrics of the laser including improved signal-to-noise ratio, reduced fabrication complexity, and enlarged tolerance. Our results may pave the way for future research on high *Q* factor lasers with large output power.

## Finite size effect

**Fig. 1:**
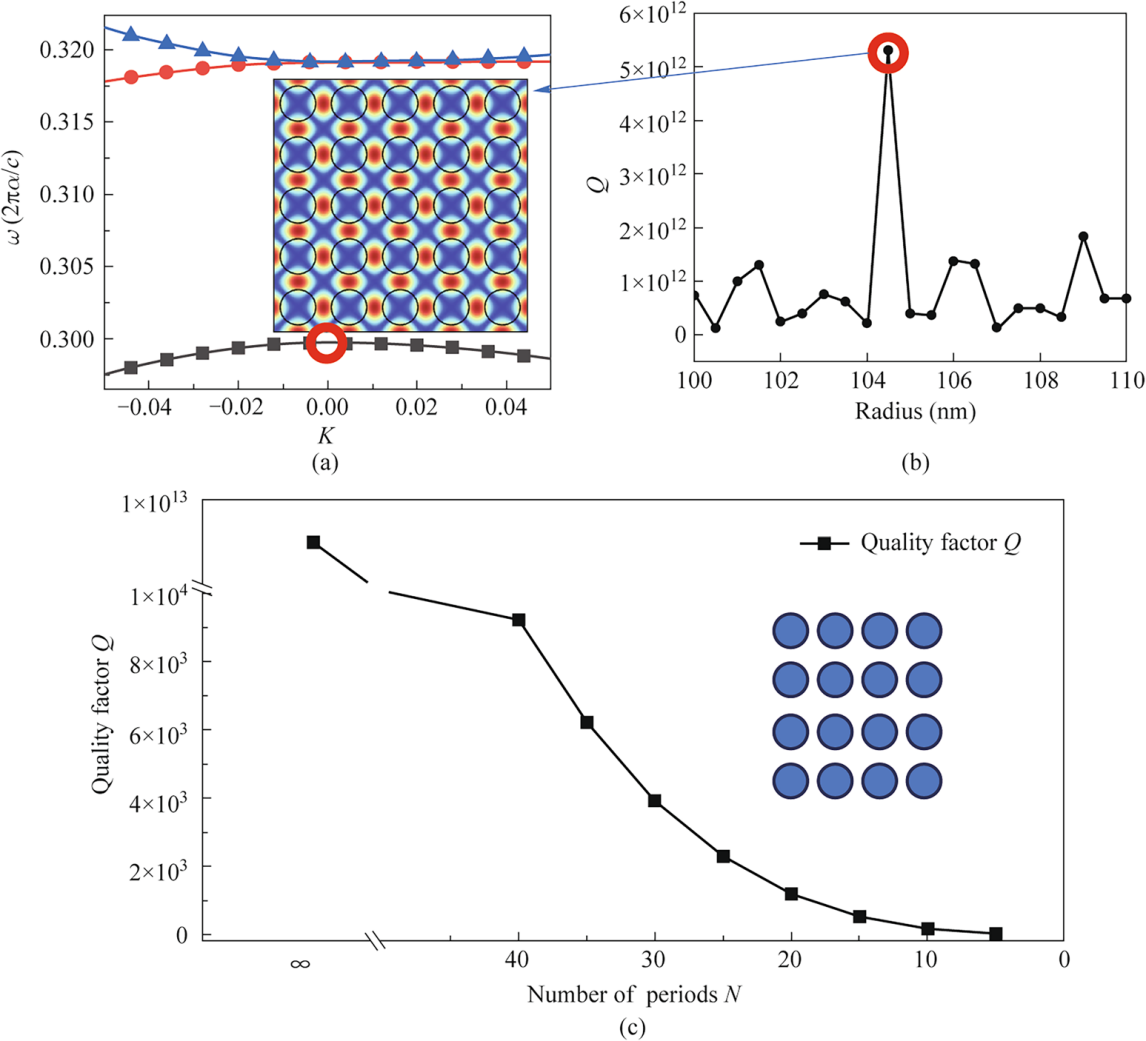
Numerical analysis of photonic crystal modes in finite-size structures. **a** Band structure for a in finite photonic crystal lattice. Insert: Simulated electric field distribution of the BIC mode. **b** Dependence of the *Q* factor on the photonic crystal hole radius, showing a pronounced resonance peak corresponding to the bound state in the continuum (BIC) condition. **c** Evolution of the quality factor *Q* as a function of the number of photonic crystal periods *N*, highlighting the dramatic drop in *Q* due to finite-size limitations

For the ideal situation, BIC occurs in a symmetrical photonic crystal with infinite size. Figure 1a depicts the band structure of a square-latticed photonic crystals where periodical boundary condition is applied both horizontally and vertically so that infinite large photonic crystal size is ensured. The modal distribution at Gamma point (${\Gamma }$=0) at the lowest band is depicted in the insert picture of Fig. 1a where a strong localized pattern is obtained in terms of a standing wave, which predicts the formation of BIC. The corresponding *Q* factor under different photonic crystal radius can be seen in Fig. 1b), where a maximum *Q* factor is achieved at ${r_0}$=105 nm. The *Q* factor rapidly declines as long as r is deviated from ${r_0}$, suggesting that the obtained extremely high *Q* factor is an ideal BIC mode. However, in practical situations photonic crystals have limited size, thus the *Q* factor may not be the same as in the maximum *Q* in Fig. 1b. To understand the finite size influence, Fig. 1c) plots the *Q* factor values when the size of photonic crystals *N* (suppose 2D photonic crystal have *N* rows and *N* columns of air holes) decrease from infinite into finite number, then gradually reduced from 40 to 5. A significant reduction is observed in Fig. 1c), as *Q* value decreases from ${5.8 \times 10^{12}}$ for infinite *N* to *Q*=${9.2 \times 10^3}$ for *N*=40, till *Q*=30.5 for *N*=5. This further proves the difficulties to obtain BIC mode in photonic crystals as photonic crystals always have finite size. In the following sections, we chose *N*=11 as a starting point because this number of air holes is most easy to fabricate, where the *Q* factor is only 186.

The significant reduction in *Q* factor for finite-sized photonic crystals can be attributed to the fundamental disruption of the BIC mode conditions at boundaries. In an infinite photonic crystal, BIC exists due to perfect destructive interference of radiation channels and precise phase matching conditions that completely suppress out-of-plane radiation. However, when the crystal size becomes finite, the abrupt termination at boundaries truncates the mode profile, breaking this delicate balance. This truncation can be understood as introducing additional wavevector components through spatial Fourier transformation, effectively perturbing the mode from its ideal BIC state where the wavevector must precisely satisfy the BIC condition. In Fig. 1c), the rapid reduction of *Q* factor is due to the wave vector has been changed from original BIC wave vector ${k_\text{bic}}$ into a truncated wave vector ${k_\text{tru}}$ that the truncated edges and out of plane loss must be taken into account:

1 $$\begin{aligned} k_\text{tru} = k_\text{bic}+\Delta k_\text{out of plane}.\end{aligned}$$

The boundary truncation acts as a perturbation that couples the ideal BIC mode to radiative channels, leading to vertical leakage of electromagnetic energy. Furthermore, the mismatch between the truncated mode profile and the ideal BIC eigen-mode results in imperfect destructive interference of radiation channels, causing additional radiation losses. The smaller the photonic crystal size, the more severe this boundary truncation effect becomes, explaining the dramatic decrease in *Q* factor as *N* reduces from 40 to 5.

## Results

The relationship between system size *N* and *Q* factor can be understood through a scaling law analysis. For finite-size photonic crystals, the *Q* factor typically follows a power-law scaling ${Q \propto N^{\alpha } }$, where ${\alpha }$ depends on the specific mode symmetry and boundary conditions. This scaling behavior arises because the mode leakage at boundaries becomes proportionally less significant as the system size increases, allowing the central region to better approximate the ideal BIC condition. To mitigate the boundary truncation effects and improve the *Q* factor in finite-sized structures, several strategies can be implemented.

### Reflective photonic crystals design

**Fig. 2:**
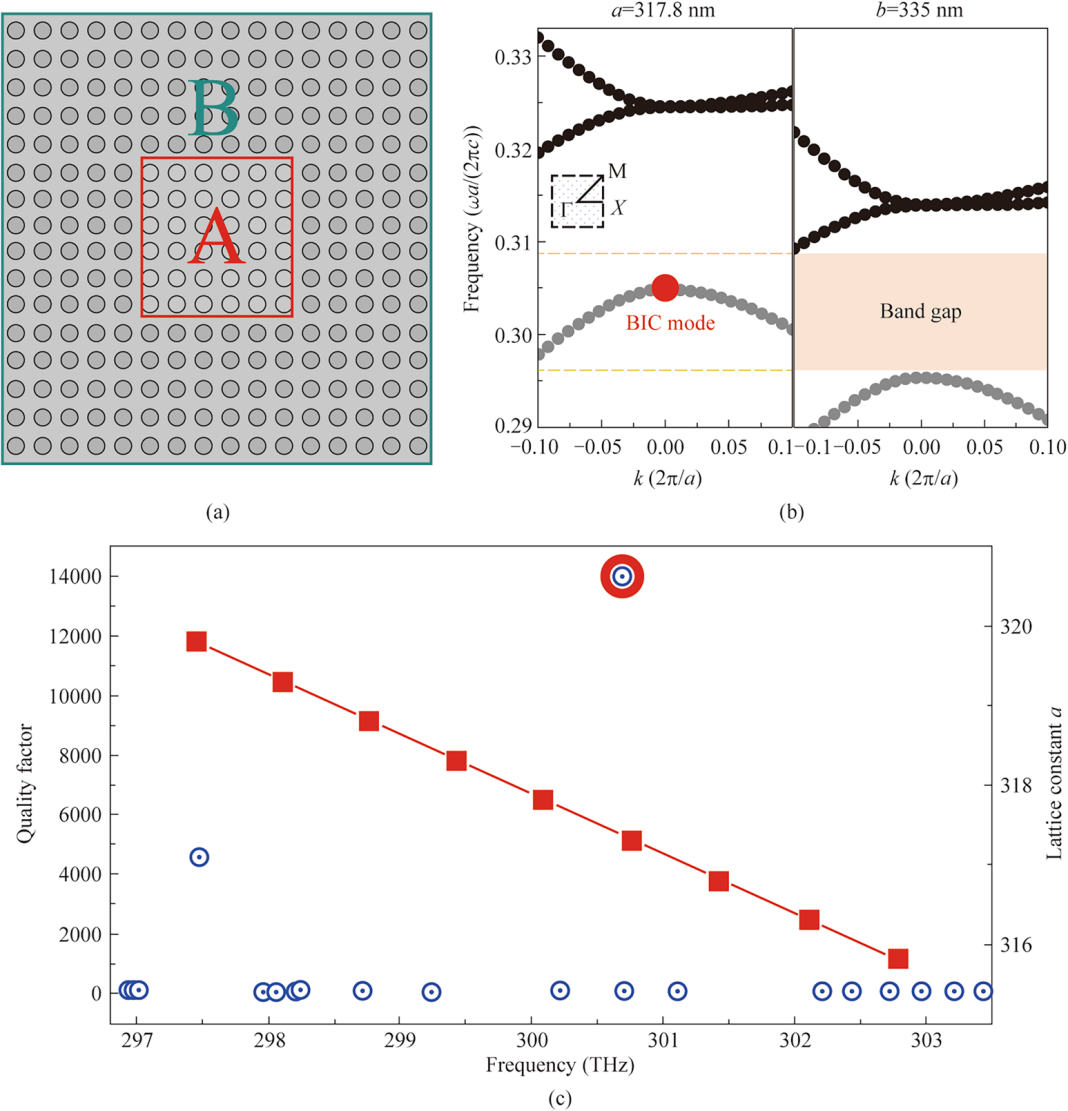
Design of BIC mode in finite photonic crystals with reflective boundaries. **a** Schematic of the proposed structure consisting of two regions: central photonic crystal A (red square) supporting BIC mode and surrounding photonic crystal B (cyan border) providing mode confinement. **b** Band structure calculated for photonic crystals with lattice constants *a*=317.8 nm and *b*=335 nm. The red dot indicates the BIC mode frequency, which falls within the bandgap (shaded region) of the surrounding photonic crystal B. **c** Left *y* axis: Quality factor as a function of frequency, showing a sharp resonance peak of *Q*=13,974 at 300.6 THz, demonstrating effective mode confinement by the reflective boundary design, right *y* axis: An example for scalability: the resonant frequency variation under different lattice constant *a* while *b* fixed at 335 nm

To mitigate the boundary truncation effects in finite-sized photonic crystals, one possible approach is to add a surrounding reflective lattice region near the edge composed of secondary photonic crystals. As shown in Fig. 2a, the proposed structure consists of two distinct regions: a central photonic crystal (region A, marked by red square) that supports the BIC mode, surrounded by a secondary photonic crystal (region B, marked by cyan border) designed to provide mode confinement. The band structures of these photonic crystals are carefully engineered, as illustrated in Fig. 2b, where photonic crystal B exhibits a complete photonic bandgap (shaded region) precisely at the BIC mode frequency of photonic crystal A for *N*=11. This bandgap alignment is crucial - the BIC mode frequency (marked as red dot) falls within the bandgap of photonic crystal B, ensuring effective reflection of the mode back into region A. However, as the period of photonic crystal A and period of photonic crystal B is different, it will be difficult to connect photonic crystal A and B smoothly without lattice mismatch. As a result, photonic crystal B is deliberately set to have the same period *a*=317.8 nm along its interface with photonic crystal A, while have different period *b*=335 nm in the direction perpendicular to the interface.

The effectiveness of this design can be quantitatively verified through the quality factor analysis presented in Fig. 2c. The *Q* factor reaches a remarkable peak value of 13,974 at the BIC frequency of 300.6 THz, representing nearly two orders of magnitude improvement compared to the *Q* factor of 186 observed in the bare finite structure with *N*=11. This dramatic enhancement can be attributed to two key mechanisms. First, the photonic bandgap of region B creates a ‘soft boundary’ that gradually reflects the BIC mode rather than causing abrupt truncation, thereby preserving the mode’s symmetry and reducing unwanted wavevector components. Second, the smooth transition between regions A and B allows for adiabatic mode adaptation, minimizing scattering losses at the interface. The sharp resonance peak in Fig. 2c further confirms that the mode maintains its high *Q* characteristics despite the finite size constraints, suggesting that the reflective boundary successfully emulates the behavior of an infinite photonic crystal.

This approach offers significant advantages over traditional edge termination techniques, as it provides a robust and physically intuitive solution to the finite-size problem. The design principles are readily scalable and can be adapted to different frequencies by adjusting the lattice parameters *a* and *b*, which are optimized here to 317.8 and 335 nm respectively. As an example in scalability, the red square line in Fig. 2c records the resonant peak frequency variation under the increase of lattice constant *a* while lattice constant *b* is fixed at 335 nm. It should be point out that changing lattice constant b will have similar effect on shit of the resonant peak frequency.

### Graded photonic crystals design

**Fig. 3:**
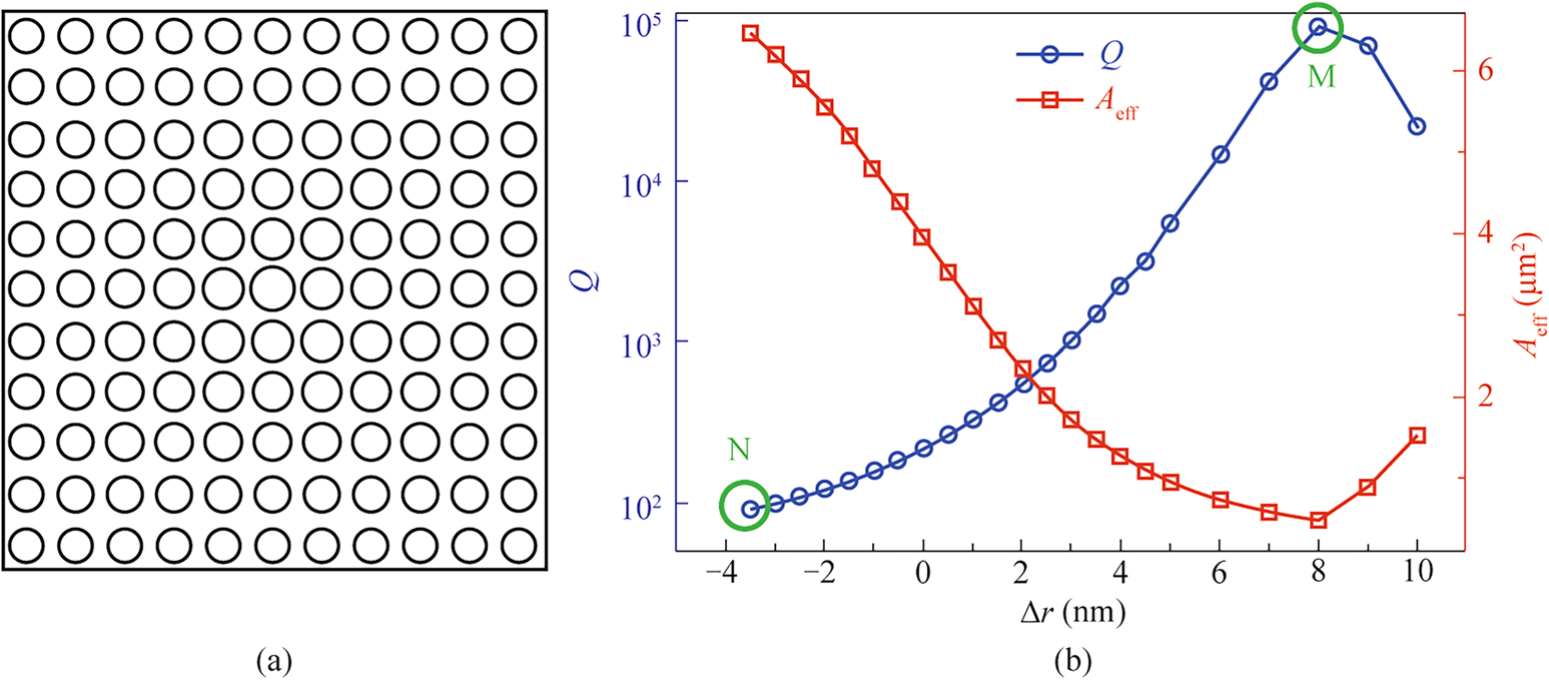
Graded photonic crystal design for adiabatic mode transition. **a** Schematic illustration of the graded photonic crystal structure where hole radii gradually vary from center to edge. **b** Quality factor *Q* (blue circles) and effective mode area $A_\text{eff}$ (red squares) as a function of radius variation $\Delta r$

To create a more adiabatic transition region that reduce the abrupt mode truncation, graded photonic crystal structure is further considered here to tail the mode performance by gradually adjusting the wave vector in each period. Figure 3a illustrate the schematic of graded photonic crystals, where the radius of the adjacent air-holes is gradually changed from the center towards the edge, following ${r_{i+1} = r_{i} + \Delta r}$. In the simulations, the first row/column of air-hole is fixed at ${r_0}=105 \text{ nm}$ as a starting point, where other row/column of air-hole are gradually increased, as depicted in Fig. 3a. This gradual variation creates an adiabatic transition region that helps to minimize mode truncation effects.

Figure 3b depicted the simulation results of quality factor *Q* variation with radius changes where from edge to the center, $\Delta r> 0$ if the air hole radius is gradually increased, whereas $\Delta r>0$ depicts the situation that the air hole radius is gradually reduced. It can be found that when radius is gradually increased, the quality factor *Q* monotonically raise up and the *Q* value is found significantly enhanced by 3 orders of magnitude until it reaches its maximum *Q*=92091 at the increase step ${ \Delta r}$ =8 nm. On the contrary, radius reduction is also investigated in Fig. 3b as the negative ${ \Delta r}$ part. It can be found that *Q* factor continuously reduces when the radius is gradually decreased from the first row/column towards the center. It should be also mentioned when radius continuous increases after $\Delta r> 8 \text{ nm}$, the *Q* factor starts to decline after its peak point, showing $\Delta r = 8 \text{ nm}$ is the optimal value for this fixed graded photonic crystal size. To understand this tendency, the modal effective area $A_\text{eff}$ is also plotted as the red rectangle dot curve in Fig. 3b), which demonstrates opposite behaviors to the *Q* factor. This is expected, as smaller $A_\text{eff}$ indicates the mode is localized stronger inside photonic crystals which is a sign for high quality factor *Q*.

**Fig. 4:**
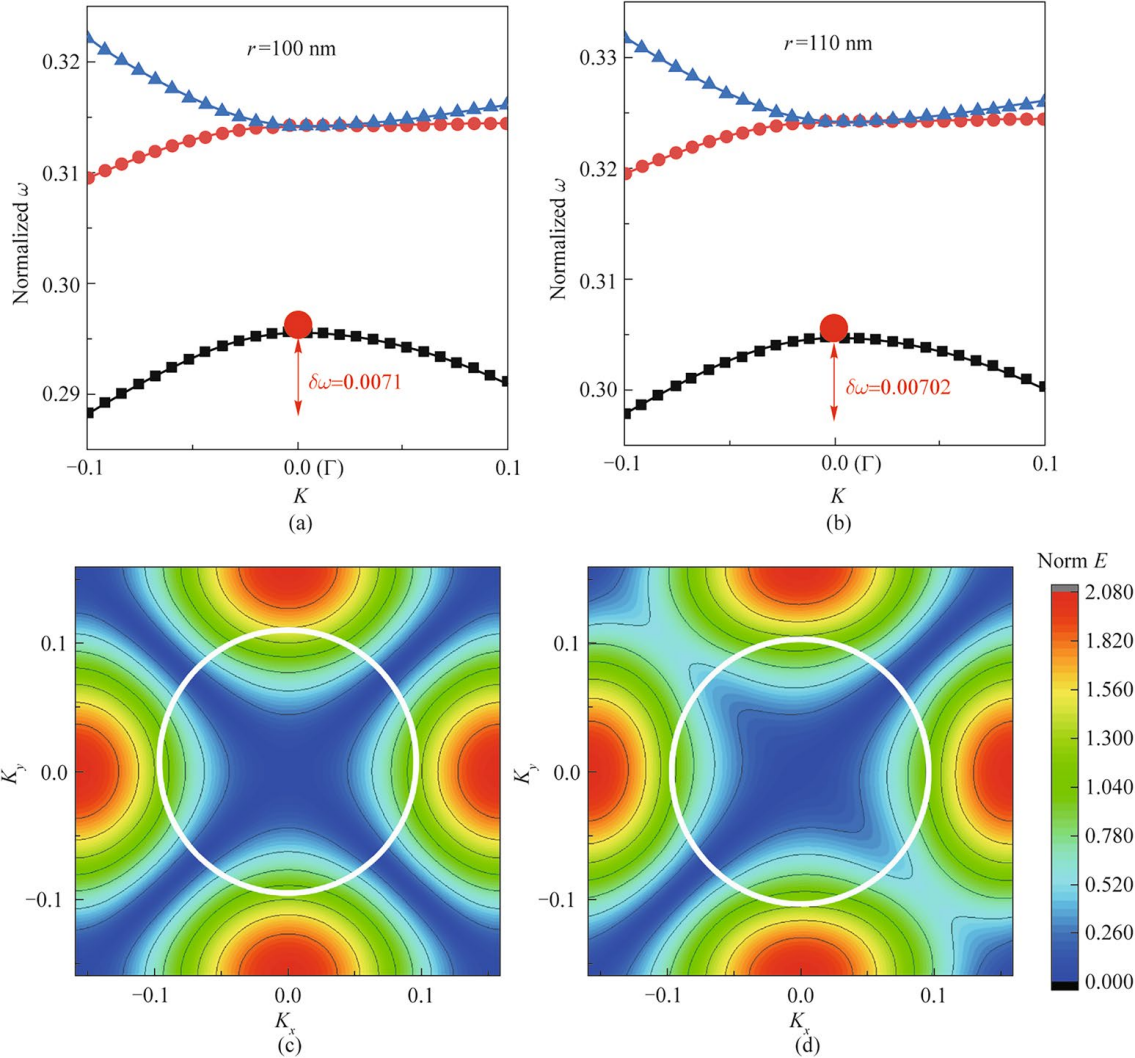
Band and mode Comparison between radius at 100 nm and radius 110 nm, **a** band structures of radius 100 nm, **b** band structures of radius 110 nm, **c** mode distribution near $\gamma$ point in the reciprocal space for radius 100 nm, and **d** mode distribution near $\gamma$ point in the reciprocal space for radius 110 nm

To get physical insight of these rapid increase of *Q* factor, Fig. 4 compares the band structures between smaller radius (*r*=100 nm, Fig. 4a) and larger radius (*r*=110 nm, Fig. 4b). It is evident when radius is enlarged all the bands move upward. For the fundamental band where the BIC mode locates, it moves upward but the band also becomes flatter as the normalized frequency reduces slower when it is away from the central $\Gamma$ point. As the BIC mode is a perfect localized mode in terms of the standing wave that corresponds to the flat band point $\frac{\textrm{d} w}{\textrm{d} k} =0$ at the peak of the band. A flatter band curve indicates a stronger localization at $\Gamma$, indicating more difficult for the mode to escape as the frequency reduces slower when *k* is away from $\Gamma$. Another evidence of this stronger confinement at large radius is the corresponding modal profiles. Figure 4c and d compare the modal distributions in the first Brillouin zone between smaller radius (*r*=100 nm, Fig. 4c) and larger radius (*r*=110 nm, Fig. 4d). Compared with the modal profile at smaller radius in Fig. 4c, the mode at larger radius turns into ellipse shape and is clearly pushed to the edge side (the high index medium region) which means the mode is more confined to the high index medium indicating an enhanced localization. For a limited size graded photonic crystals like *N*=11 in Fig. 3a, the radius is larger in the center so that the confinement in the central region is the stronger; while towards the edge sides the mode is less confined. and at the open boundary outside of graded photonic crystals there is no confinement. As a result, by gradually increasing the radius of the air hole from edge towards center, the mode is gradually transitioning from weak confinement into a stronger confinement, which not only change the wave vector gradually from open boundary into BIC mode, but also avoid the strong mode mismatch (in terms of out of plane loss) at the truncated edge of the boundary. Furthermore, it can be also explicated via the wave vector. As previously explained in Eq. (1), the rapid reduction of *Q* factor is due to the wave vector has been changed from original BIC wave vector ${k_\text{bic}}$ into a truncated wave vector ${k_\text{tru}}$ that the truncated edges and out of plane loss must be taken into account. However, when graded photonic crystals are applied, the wave vector $k_\text{grad}$ also consider the gradually changed wave vector introduced by the radius change $\Delta k_{\text{radius}\_\text{modulate}}$ across each of the transition period:

2 $$\begin{aligned} {k_\text{grad} = k_\text{bic}+\Delta k_{\text{out}\_\text{of}\_\text{plane}}+\Delta k_{\text{radius}\_\text{modulate}}} \end{aligned}.$$

When radius is gradually increased from edge toward the center, the $\Delta k_{\text{radius}\_\text{modulate}}$ is on the opposite sign of $\Delta k_{\text{out}\_\text{of}\_\text{plane}}$, which happens to mitigate the truncated edge effect due to the finite size.

**Fig. 5:**
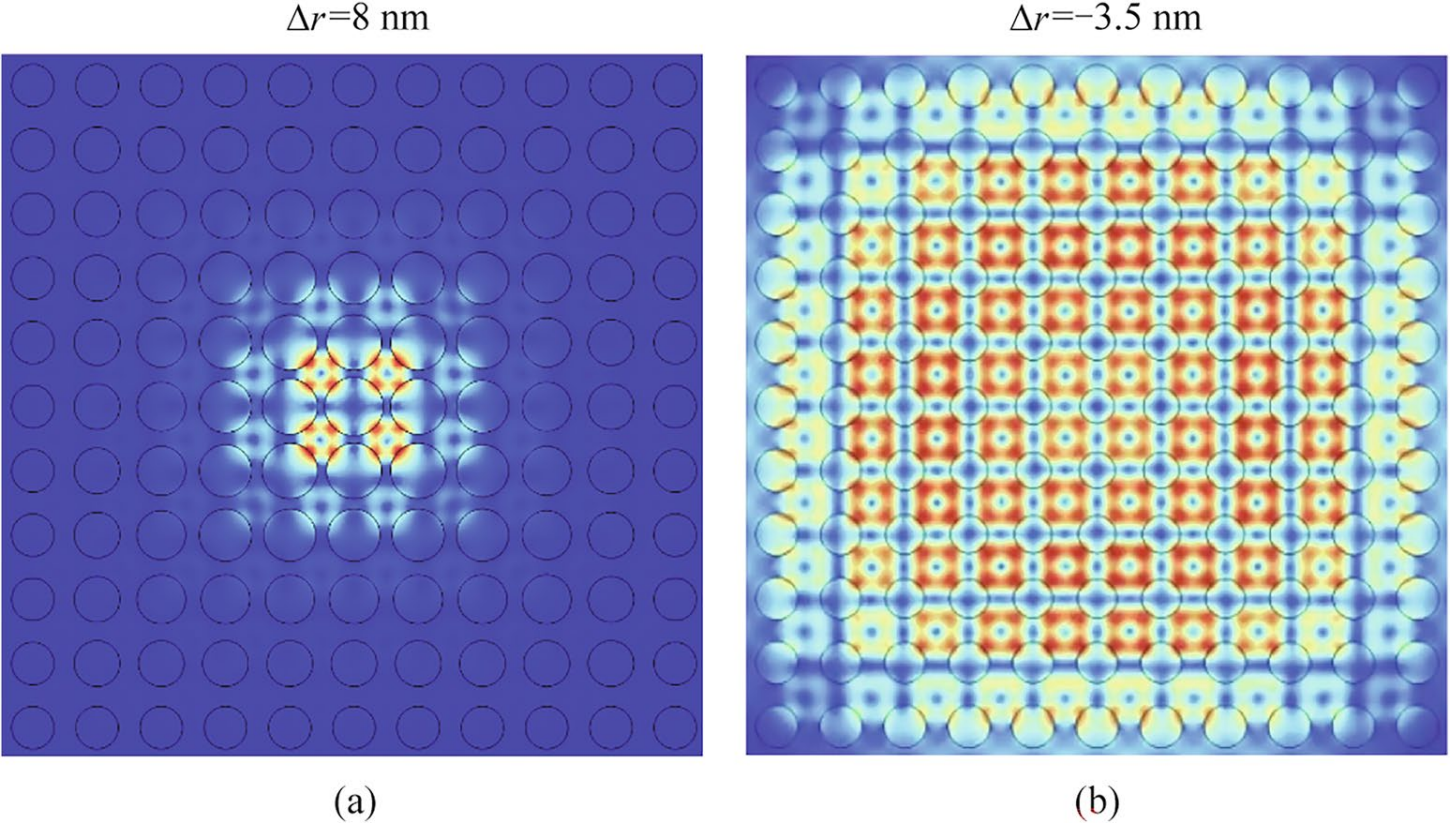
Electric field distribution in graded photonic crystals with different radius variations. **a** Mode profile for $\Delta r$ = 8 nm showing strong spatial confinement with exponential field decay. **b** Mode profile for $\Delta r$ = $-$3.5 nm exhibiting extended field distribution with weaker confinement

To better understand how the graded photonic crystal affects modal confinement and *Q* factor, Fig. 5 depicts two extreme cases of graded photonic crystals: one with a positive radius gradient and the other with a negative radius gradient to show the contrast. The first case is for the Positive Radius Gradient ($\Delta r =8 \text{ nm}$ ). As shown in Fig. 5a, the modal distribution corresponds to the M point in Fig. 3b. In this scenario, the optical field is tightly confined in the central region of the photonic crystal, staying far away from the structure’s boundaries. This behavior occurs because the graded photonic crystal with a positive radius gradient creates a buffer layer that isolates the optical field from direct interaction with the boundaries. As a result, radiation losses caused by boundary scattering are effectively eliminated. This explains why positive radius gradients ($\Delta r>0$) provide stronger mode confinement and higher *Q* factors. The second case is for the negative Radius Gradient. Figure 5b shows the modal distribution when $\Delta r =-3.5 \text{ nm}$ corresponding to the *N* point in Fig. 3b. In this case, the optical field spreads extensively across the structure, reaching the boundaries where it experiences abrupt truncation. This truncation leads to significant radiation losses, resulting in a much lower *Q* factor. The lack of a smooth transition at the boundaries in this design exacerbates scattering and radiative losses.

The comparison between these two cases demonstrates that a positive graded design effectively improves the *Q* factor by providing better mode confinement and minimizing radiation losses at the boundaries. This highlights the importance of positive radius gradients in achieving high *Q* characteristics in graded photonic crystals.

## Device fabrication and characterization

**Fig. 6:**
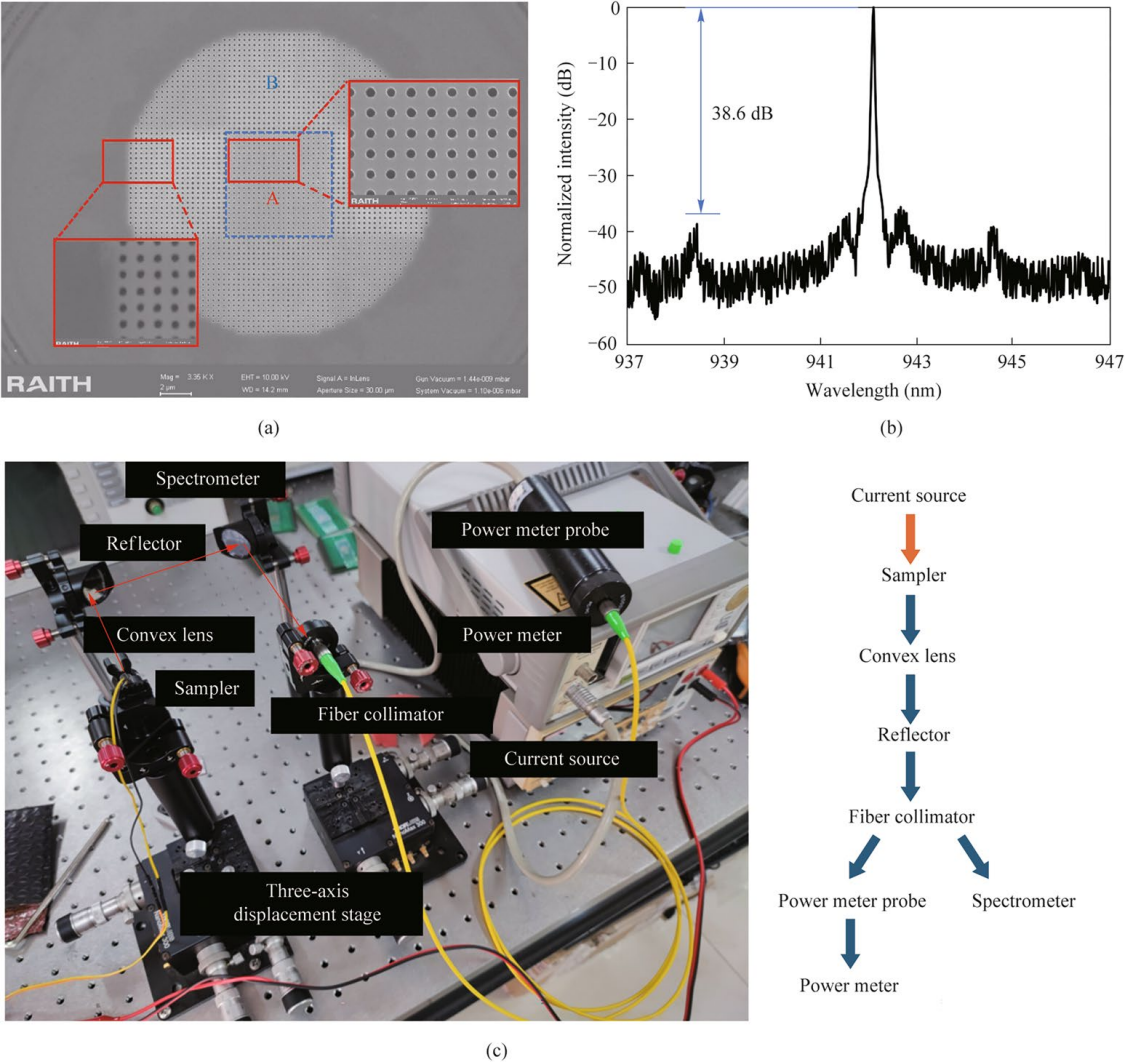
Device fabrication and characterization of reflective photonic crystal cavity laser. **a** Scanning electron microscope image after the E-beam lithography process, showing the multi-layer structure consisting of two different lattice constants photonic crystals for region A and region B, Inset: magnified view of the photonic crystal holes showing high-quality pattern definition. **b** Measured emission spectrum of the microlaser demonstrating single-mode operation with a signal-to-noise ratio of 38.6 dB. **c** Experimental setup for optical characterization including precision current source, three-axis stage, and optical measurement system for both spectral and power analysis

Although the graded photonic crystal cavity approach has higher *Q* factor, it requires extreme accuracy of radius size adjustment ($\Delta r$=8 nm) which is quite challenging for our E-beam lithography process. In the fabrication process, the reflective photonic crystal cavity approach is chosen to be fabricated as it is particularly advantageous because it offers a straightforward implementation that avoids the complexity of precise control engineering of graded radius while providing robust performance improvements. The fabrication began with spin-coating ZEP520 electron-beam resist onto the substrate, followed by a systematic three-step aligned electron-beam lithography process. First, the mesa pattern was defined and etched, providing the foundational structure. Subsequently, the photonic crystal pattern was carefully aligned and fabricated, followed by the final step of electrode patterning. The reactive ion etching (RIE) parameters were meticulously optimized for different feature sizes to ensure precise control over the hole dimensions and sidewall profiles, which are critical for maintaining high *Q* factor performance. The photonic crystal microcavity was fabricated through a precise multi-step electron-beam lithography and etching process. As shown in Fig. 6a, the device consists of three crucial components: the photonic crystal pattern with lattice constant of 317.8 nm in region A, the second photonic crystal pattern with lattice constant of 335 nm in region B, and mesa.

The scanning electron microscope (SEM) image in Fig. 6a reveals the high quality of the fabricated structure, showing well-defined photonic crystal holes and clear device geometry. The contrast between regions A and B demonstrates successful implementation of our multi-step fabrication strategy, with precise alignment between different layers. The zoom-in view (red inset) shows the detailed photonic crystal pattern with excellent uniformity and circularity of the holes, which is crucial for achieving the designed optical properties.

The laser emission from our photonic crystal microcavity is achieved through careful design of the vertical structure using distributed Bragg reflectors (DBRs). The DBR mirrors consist of periodically stacked alternating layers of high and low refractive index materials, where each layer is designed with a quarter-wavelength optical thickness to maximize reflectivity at the target wavelength. We used DBR sampler fabricated by Vertilite Co., Ltd, formed by P-doped and N-doped DBR mirrors on the top and bottom surfaces, respectively. An asymmetric design, where the P-DBR has fewer pairs than the N-DBR, creates an intentional reflectivity difference between the top and bottom mirrors. Consequently, laser emission occurs preferentially through the P-DBR surface, ensuring directional output from the device. This vertical cavity design, combined with the lateral confinement provided by the photonic crystal structure, enables efficient single-mode laser operation. In Fig. 6b, the laser exhibits a sharp emission peak with a signal-to-noise ratio of 38.6 dB, indicating high-quality single-mode operation. The background noises are residual feedback from the optical components, not the intrinsic output due to the laser cavity’s emission, which are observed in Fig. 6b at a level of more than 45 dB. However, we rigorously choose the secondary high peak as background noise under which an extinction ratio of 38.6 dB can be obtained.

The optical characterization setup is illustrated in Fig. 6c, where the optical output was simultaneously monitored through two measurement channels: one leading to a power meter for precise output power measurements, and another connected to a high-resolution optical spectrum analyzer for spectral analysis. This dual-measurement capability allows for concurrent monitoring of both spectral purity and output power stability. The photonic crystal microlaser was mounted on a precision three-axis translation stage for optimal alignment control. Electrical pumping was achieved through a high-precision current source with remarkable resolution (1 pA/100 nV), enabling fine control over the injection current. The emitted laser light was collected through a specially designed optical path consisting of a lens system that collimates the output beam, followed by efficient coupling into an optical fiber through a precision fiber collimator.

**Table 1:** Comparison of laser parameter performance

	Pump type	Wavelength (nm)	Lattice constant (nm)	Hole radius (nm)	Quality factor
Ref. [11]	Optical	1550	568	200	$\sim 8000$
Ref. [6]	Electric	100000	35000	9700	2915
Ref. [18]	Electric	940	5000	1250	6238
This work	Electric	940	317.8	105	11776

Table 1 presents a comprehensive comparison between our work and previously reported photonic crystal lasers, demonstrating several significant advances. Most notably, our device represents the first demonstration of an electrically-pumped BIC laser operating at the 940 nm wavelength band. While previous works have achieved electrical pumping at either much longer wavelengths or similar wavelengths without BIC designs, our implementation uniquely combines electrical pumping capability with BIC mode operation in this technologically important wavelength regime. As a result, our work achieves the highest quality factor (11,776) among electrically pumped devices, representing nearly a two-fold improvement over Ref. [14] and a four-fold enhancement compared to Ref. [13]. This superior *Q* factor is particularly noteworthy considering our substantially more compact design, featuring the smallest lattice constant (317.8 nm) and hole radius (105 nm) among all compared structures.

A particular challenge of our design is the remarkably small feature size required for BIC mode formation at 940 nm. The miniaturization of our device, while maintaining high performance, represents a significant advancement in photonic crystal laser design. Notably, while Ref. [18] operates at 1550 nm with optical pumping, our device achieves comparable *Q* factor performance at 940 nm with electrical pumping, which is generally more challenging due to additional loss mechanisms associated with electrical injection. Furthermore, our design has significantly reduced the cavity size through optimization if compared with the design in Refs. [8, 18], providing a more in-depth analysis for the size dependence of BIC cavities. The compact dimensions of our design (lattice constant = 317.8 nm) compared to other electrically pumped devices (Refs. [13] and [14] with lattice constants of 35000 and 5000 nm, respectively) demonstrates superior integration potential for photonic integrated circuits.

Furthermore, our design achieves superior optical confinement as evidenced by the highest *Q* factor (11,776) among comparable simple-structured photonic crystal lasers. This enhanced *Q* factor, notably higher than previous reports, is achieved without resorting to complex designs such as nested photonic crystals or irregular lattice patterns [19]. This remarkable performance in a straightforward structure highlights the effectiveness of our BIC-based design approach, demonstrating that proper engineering of symmetry-protected BIC modes can yield superior performance while maintaining structural simplicity and fabrication feasibility.

## Conclusion

In this work, we have comprehensively investigated and demonstrated effective strategies to overcome the finite-size limitations of bound states in the continuum (BIC) in photonic crystal structures. Two distinct approaches were proposed and successfully demonstrated: *Q*=13,974 and *Q*=92,091 are obtained in the reflective boundary photonic crystal design and the graded photonic crystal design, respectively. Experimental verification for the reflective boundary photonic crystal design was demonstrated with a high signal-to-noise ratio of 38.6 dB, validating the effectiveness of our design approach in practical applications. The obtained results may pave the way for future design of BIC with finite size in real applications, and promote the performances of integrated laser based on BIC effects.

## Data Availability

Data underlying the results presented in this paper are not publicly available at this time but may be obtained from the authors upon reasonable request.
